# Implementing an interprofessional point-of-care ultrasound protocol for dyspneic patients in an emergency department as a blended learning concept—Feasibility of Employing Thoracic Ultrasound in Shortness of Breath

**DOI:** 10.3389/fmed.2023.1193243

**Published:** 2023-08-22

**Authors:** Matthies Witte, Matthias Ott, Tobias Schilling, Martina Müller, Stephan Schmid, Alexander Krohn

**Affiliations:** ^1^Department of Interdisciplinary Acute, Emergency and Intensive Care Medicine (DIANI), Klinikum Stuttgart, Stuttgart, Germany; ^2^Department of Internal Medicine I, Gastroenterology, Hepatology, Endocrinology, Rheumatology, and Infectious Diseases, University Hospital Regensburg, Regensburg, Germany

**Keywords:** dyspnea, point-of-care ultrasound, RADiUS protocol, interprofessional, emergency medicine, sonography

## Abstract

**Objective:**

Dyspnea is a common symptom in the Emergency Department, with a wide variety of differential diagnoses. Previous research has demonstrated the diagnostic accuracy of Point-of-Care Ultrasound (POCUS) in this field of interest. Our goal was to better establish sonography in our emergency department with a practicable and time effective method. Therefore, we implemented a sonography protocol in an interprofessional emergency team using blended learning as a modern didactic approach and evaluated the learning and teaching success. We named the study FETUS, which stands for “Feasibility of Employing Thoracic Ultrasound in Shortness of Breath.”

**Methods:**

A demonstration of the POCUS protocol was given, followed by individual supervision during clinical routine. A written manual, a pocket card, and further materials for personal training supplemented the training. A post-training questionnaire measured several parameters regarding the training, e.g., subjective skill-acquisition or media use.

**Results:**

32 medical and nursing staff participated in this study, 14 of whom completed the questionnaire. All training modalities offered were well received. A pre-post comparison of subjective sonographic competence shows a significant increase in both medical and nursing staff.

The other items surveyed also indicate the success of the intervention undertaken.

**Conclusion:**

The use of different media as a blended learning approach can support the implementation of new measures in the ongoing working routine within an interprofessional team.

## 1. Introduction

Sonographic examination of the lung has been studied for several decades (1–6). Traditionally, it was thought to be of little use due to the sound-reflecting nature of subpleural air, which limits the sonographic visibility to only a few centimeters in depth. Other imaging modalities with greater penetration, such as chest radiography, have long been the gold standard (7–11). Although the physical nature of lung ultrasound is undisputed, the significance of the pleural processes visible on ultrasound for the entire lung has been well established by numerous studies, since most.

pneumological pathologies relevant to emergency department also involve peripheral lung segments (12–18). Pulmonary ultrasound has been shown to be highly effective in a wide variety of lung diseases, in most cases superior to chest radiography. This includes diagnoses such as pulmonary edema, pneumonia, acute respiratory distress syndrome, consolidations, or pneumothorax (19–24). Certainly, the global pandemic of SARS-CoV-2 with a high rate of respiratory manifestations and a temporary lack of adequate diagnostic equipment was a catalyst for the development of lung ultrasound as a rapid and powerful examination modality (25–29).

According to various studies, dyspnea is one of the three most common chief complaints in the emergency department (30). The variety of possible pathologies that can cause respiratory distress poses a challenge to rapid medical evaluation. Since not only pulmonary but also cardiovascular or hematological disorders can cause dyspnea, a multitude of differential diagnoses must be considered (31). Clinical differentiation between cardiac and pulmonary etiologies of acute to chronic deterioration of respiratory symptoms is often challenging even for experts. Recent technological advances in making ultrasound smaller and more portable have had a tremendous impact on establishing a bedside application (32), both for physicians and nurses. Systematically performing this Point-of-Care-Ultrasound (POCUS) examination as an adjunct to the medical history and physical examination can significantly improve the medical decision-making process by allowing the examiner to differentiate an unclear etiology early and to understand pathophysiological mechanisms, leading to appropriate treatment (33, 34).

Due to the complexity of the organ systems involved in adequate oxygenation of the organism, the sonographic protocols used must take these conditions into account. A detailed examination of the lungs alone would not be sufficient for this purpose, nor would be focused echocardiography. Following established emergency medicine schemes such as the FAST (*Focused Assessment with Sonography for Trauma*) or RUSH (*Rapid Ultrasound in Shock and Hypotension*), the “Rapid Assessment of Dyspnea with Ultrasound” (RADiUS) by Manson and Hafez includes not only the pleural and echocardiologic examination, but also the inspection of the inferior vena cava (IVC) and the pleural cavity (35). This approach of examining these four components as a compromise made between an extensive screening of dyspneic patients and the time-efficient approach for emergency medicine routines. Lamsam et al. modified the RADiUS protocol by adding the short-axis view to the echocardiography and the eight Volpicelli lung zones to the pleural examination (36, 37). This increases the number of detectable differential diagnoses is increased due to a more precise and focal inspection of the pleural processes, making the modified RADiUS protocol a profound scheme for the sonographic work-up of dyspnea.

Based on the above considerations, our goal was to scientifically investigate the systematic implementation of this protocol and its impact on clinical practice. We have established a teaching program and implemented it under the acronym FETUS, which stands for “Feasibility of Employing Thoracic Ultrasound in Shortness of Breath.” Because nurses are the first point of contact with emergency patients and play an important role in interpreting symptoms and initiating diagnostics, we implemented the protocol interprofessionally in both professional groups, nurses, and physicians. Given the already documented efficacy of thoracic ultrasound, a relative large number of ED staff had to be trained to perform an adequate number of sonographic exams. To achieve a high response rate, the training concept had to be adapted to the challenging working conditions of clinical emergency medicine such as the high workload, shift work, heterogeneous level of prior knowledge of nurses and physicians, and hygiene regulations due to the COVID-19 pandemic. As part of this process, a multimodal teaching concept was developed that incorporated different didactic approaches to address the interprofessional challenges mentioned above. Blended learning was originally described as an educational method that combines face-to-face lessons with asynchronous teaching units (38). Staker and Horn provide a more nuanced definition of blended learning as “a formal education program in which a student learns at least in part through online delivery of content and instruction with some element of student control over time, place, path and/or pace; and at least in part at a supervised brick-and-mortar location away from home” (39). In addition to meeting students’ needs for greater flexibility in learning new content and a partial detachment from local presence, this approach also allows for more specific personalization of teaching methods (40, 41).

The purpose of this paper is to outline our approach to teaching staff in a practical and effective way while working in daily clinical routine. We focus our efforts not only on physicians, but also interprofessional on nurses. Already today, nurses are being delegated a wide range of specific tasks, even involving ultrasound devices such as ultrasound-guided placement of peripheral venous catheters.

Overall, we hope that the methods used can help other institutions with specific training, even if not related to emergency medicine or Point-of-Care-Ultrasound.

## 2. Methods

### 2.1. Participants and study design

This study was conducted at a maximum care hospital in Stuttgart, Germany. Before the start of the study, all aspects of the research project were approved by the ethics committee of the University of Heidelberg. Thirty-two employees participated voluntarily and gave both verbal and in written consent to attend the study, representing 49.2% of the full-time positions of the ward. The ultrasound knowledge of the participants was recorded by self-assessment in the questionnaire. For the study, both medical and nursing staff were presented with two videos, each approximately 6 min in length. The videos served as an introduction to the project and provided an initial overview of the topic (42, 43). Access to the videos with QR codes distributed in the emergency department. For about a month, interested staff were invited to attend face-to-face training sessions several times a week on the premises of the emergency department.

Due to lower patient volumes in the early shift, the teaching sessions were offered after the morning briefing. The session lasted up to 45 min and included an average of three to six participants. All ultrasound training sessions were led by a single POCUS instructor, who demonstrated the POCUS exam on one of the participants. The initial training was followed by the individual instruction in the clinical routine for the specific examination, which required multiple trainers (qualified instructors, attending physicians) and a cumulative time commitment of 30 to 120 min per participant. In some cases, individual instruction extended beyond this time. This in-depth support was provided to one or two participants at a time, depending on their prior experience and level of knowledge. While only detailed questions were discussed with experienced personnel, the repetition of sonographic basics was an important aspect for inexperienced people. The latter was especially relevant for nurses, who naturally needed closer supervision due to the expansion of their original scope of practice.

This procedure allowed for the consolidation of newly acquired knowledge and the clarification of questions. The learning phase, or as we call it intervention phase, lasted 6 months. The instructor had a weekly expense of about 4 to 5 h. The instructor himself completed several DGIIN (German Society of Medical Intensive Care and Emergency Medicine) certified POCUS courses, furthermore the instructors were trained by two senior physicians with DEGUM course instructor experience and completed DEGUM (German Society for Ultrasound in Medicine) emergency sonography course. The entire teaching process was closely supervised by the senior physicians.

Training and ultrasound examinations were performed on two GE Logiq-e (GE Healthcare, Wauwatosa, WI, United States) and one Philips CX50 (Philips Healthcare, Andover, MA) ultrasound machines.

Following this training period, participants were asked to complete a post-training questionnaire, using Likert-Scale. Only one survey was conducted, which included pre- and post-training questions. As with general study participation, completion of the questionnaire was based on voluntary participation. The survey was divided into five parts according to the underlying topic: in addition to assessing subjective competence in performing a general or thoracic ultrasound before and after the training intervention, questions were asked about the general learning process, the use of different media formats, the learning environment and future independent application of the learned content. The Grazer Evaluation Model of Competence Acquisition (*Grazer Evaluationsmodell des Kompetenzerwerbs, GEKo*) was applied, developed and validated by Paechter et al. (44, 45) was used to determine the personal knowledge gain and the quality of the media-based training. The survey assessed each participant’s subjective learning success using a six-point Likert scale (1 = strongly disagree to 6 = strongly agree) to avoid a neutral position and a five-point scale (1 = strongly disagree to 5 = strongly agree) to Evaluating Media-based [according to Paechter et al. (44, 45)]. Participants also had the opportunity to formulate deficits or praise in free text. The questionnaire was administered online; a complete list of the items surveyed is presented in Table 1. In addition to the questionnaire, participants’ progress was tracked through personal supervision. No other measures were used to assess the progress of the participating medical staff.

**Table 1:** Items of the post-training questionnaire, sorted according to category.

Skill acquisition (45)	1. I now have a broader knowledge of the subject.
	2. I can give a good overview of the contents of the course.
	3. I have learned to make connections between subjects.
	4. I have learned to recognize complex connections within the subject area.
	5. My level of knowledge is now much higher than at the beginning of the semester.
Media use (44)	1. I believe that the media resources used enable a better division of the learning material.
	2. I think that the media-based course encourages interdisciplinary thinking.
	3. I think the media-based preparation supports individual learning processes.
	4. I think the online resources promote independent learning.
	5. I find that the media-supported course enables me to check my own learning progress.
	6. I find that independent learning from home is supported by the resources provided.
	7. I find that the additional online resources promote the practical relevance.
	8. The media-supported courses give me a good overview of the subject.
	9. I find that the online offerings encourage a critical examination of the content.
	10. I think the online materials promote networked thinking.
	11. I think the self-tests reflect my personal learning progress well.
Environment	1. I felt well guided.
	2. There were enough opportunities to participate in the training.
	3. There were sufficient opportunities for questions.
	4. The training was compatible with my work schedule.
	5. I think the amount of time was appropriate.
	6. I felt overwhelmed with the RADiUS training.
	7. I felt underchallenged by the RADiUS training.
	8. I was motivated to learn by the RADiUS training.
	9. I was able to bring in my previous experience.
	10. The intellectual level was appropriate.
Independent use	1. I will continue to use the skills I have learned in RADiUS examination after the study.
	2. I have the confidence to independently evaluate RADiUS findings of patients with dyspnea.

### 2.2. Further practical guides

Supplemental information was provided to allow for ongoing and asynchronous training and reference. Written explanations with illustrated examples of the RADiUS protocol were implemented into an existing IT database of departmental Standard Operating Procedures (SOPs). In addition to access on the department’s IT system, remote options from mobile and private devices were established.

**Figure 1:**
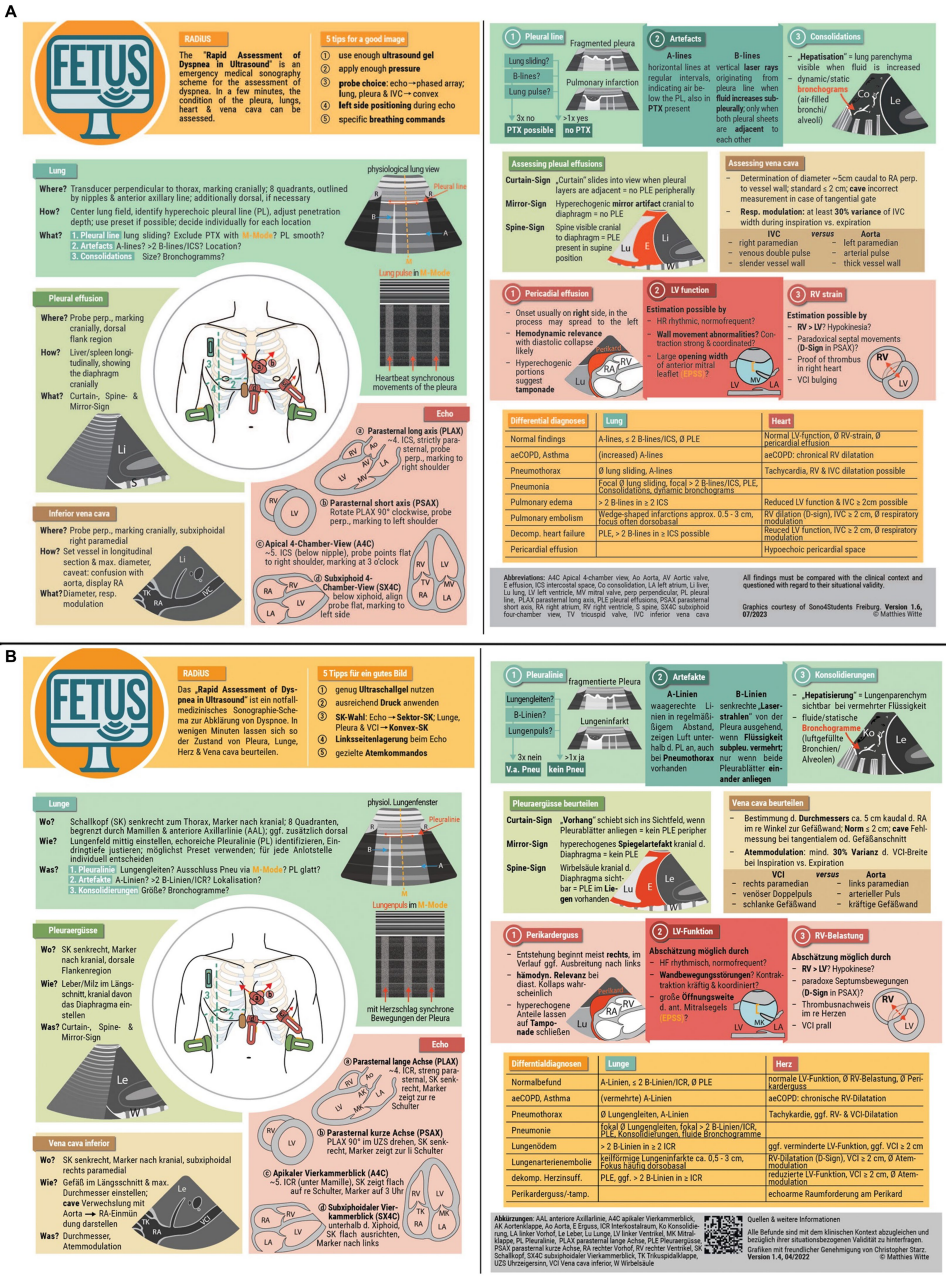
**(A)** Pocket card English (translation). **(B)** Pocket card German (original).

Serval studies have demonstrated a solid consistency in the visual assessment of cardiac function (46, 47). To support the acquisition of the necessary experience, an online learn-quiz was developed to train for echocardiographic orientation and an assessment of pump function in a variety of image and video examples. A score was used to provide feedback on personal performance. Incorrectly answered questions were explained with detailed comments. In addition, a customized pocket card was developed that can be carried at all times. It contains a brief summary of the examination procedure and possible pathologies (see Figure 1). In addition, a QR code provides access to the more detailed written explanations.

### 2.3. Statistical analysis

The questionnaire was collected anonymously, and each participant was randomly assigned an identification number, making traceability impossible. Data were analyzed and evaluated using SPSS version 28.0.1.1 (SPSS Inc., Chicago, United States; RRID:SCR_002865). A Wilcoxon matched-pairs signed rank test was applied to evaluate the difference between the pre- and post-training survey questions. A Mann–Whitney test was used to compare the professions as independent samples. Further, survey results are presented descriptively (mean, standard deviation). Survey questions on specific topics were grouped for analysis (skill acquisition, use of media formats, learning environment and independent use of learned content). *p*-values of <0.05 were considered significant.

## 3. Results

### 3.1. Overall performance

The RADiUS protocol was performed and documented in 550 patients with dyspnea over a 6 months period. A total of 52 individuals attended the training sessions offered, including seven medical students who were not enrolled in the study. Of the remaining 45 participants, 32 (61.5%) decided to continue participation in the study. The video footage was viewed a total of 178 times, the written manual a total of 196 times. Over 300 copies of the pocket cards were distributed.

### 3.2. Participant survey data

The subsequent survey was completed by 14 of the 32 participants (43.8%). Table 2 shows the age distribution, profession and professional experience in emergency medicine of the survey participants. Regarding prior experience, 26% (general ultrasound) and 13% (thoracic ultrasound) of participants reported an elevated competency measure (≥4 on the Likert scale) before the intervention was performed.

**Table 2:** Age distribution, profession and professional experience of survey participants (*n* = 14).

Profession	%	Age distribution	%	Experience in EM	%
Medical Doctor	21.4	20–30 years	42.9	<1 year	35.7
Resident	35.7	30–40 years	42.9	1–2 years	7.1
Nurse	42.9	40–50 years	7.1	3–5 years	21.4
		>50 years	7.1	5–10 years	21.4
				>10 years	14.3

**Figure 2:**
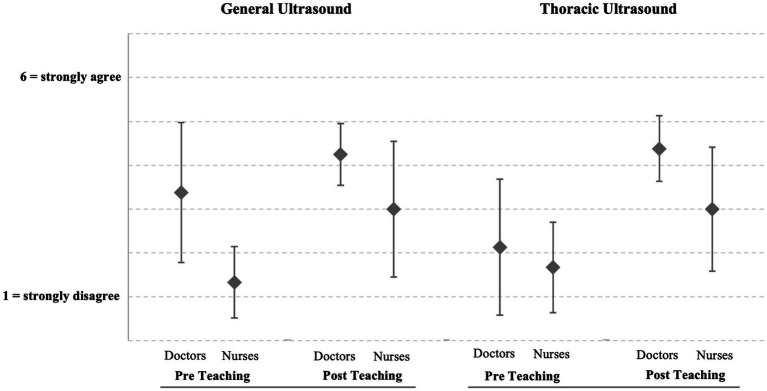
Changes in subjective competence, grouped according to profession (*n* = 14). Skills in general ultrasound before and after teaching for doctors (3.38 ± 1.60; 4.25 ± 0.72; *p* = 0.063) and nurses (1.33 ± 0.82; 3.00 ± 1.55; *p* = 0.031). Skills in thoracic ultrasound before and after teaching for doctors (2.13 ± 1.55; 4.38 ± 0.74; *p* = 0.008) and nurses (1.67 ± 1.03; N 3.00 ± 1.41; *p* = 0.063).

Subjective competence in general and thoracic ultrasound increased significantly with teaching (*p* < 0.007 and *p* < 0.002 respectively, Wilcoxon matched-pair rank test). Means, including standard deviation, are shown in Figure 2. Pearson’s correlation coefficient r was used to calculate the effect size of the change in subjective competence gain. For the use of general ultrasound, Pearson’s r is 0.49, describing a moderate effect of change, and for thoracic ultrasound, it is 0.55, describing a strong effect.

**Figure 3:**
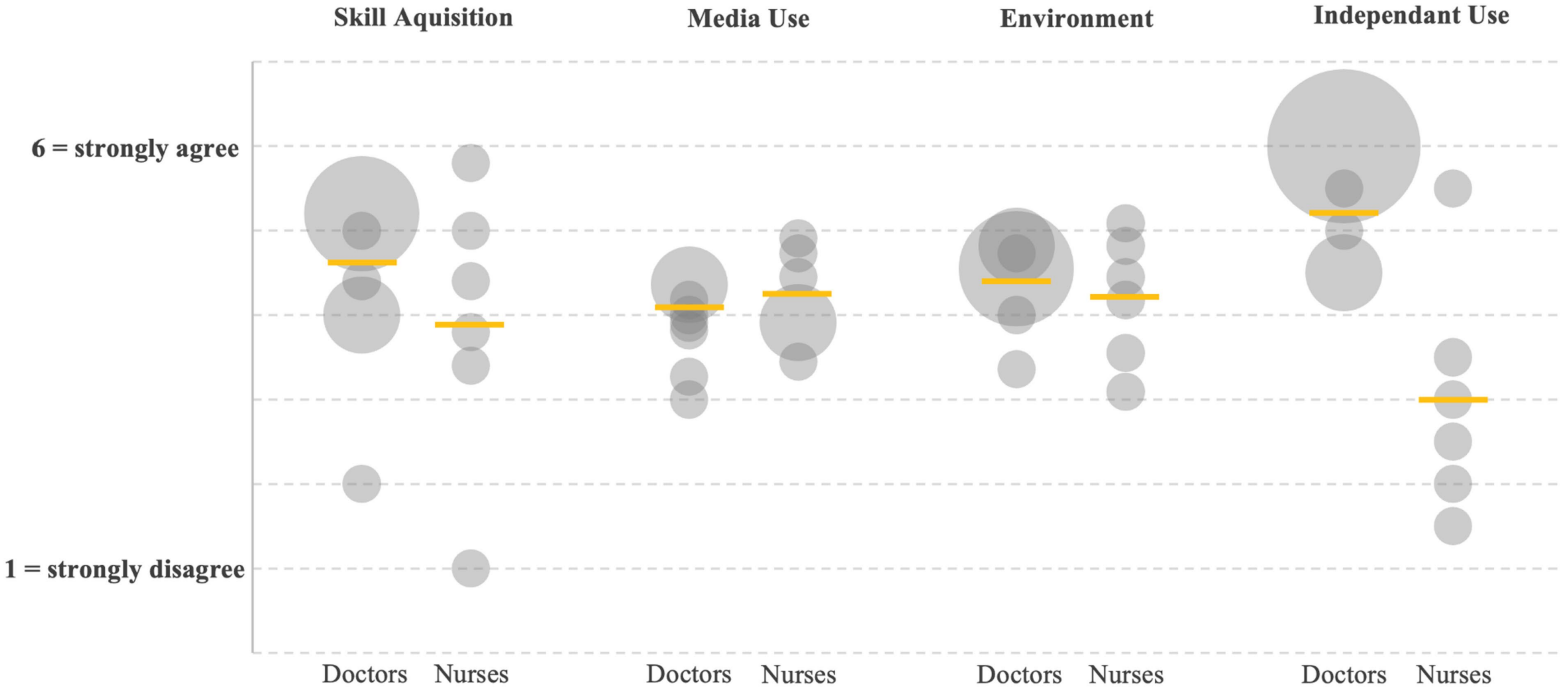
Survey results (*n* = 14), grouped according to topics and profession. (Skill Aquisition) Skill acquisition (Doctors 4.60 ± 0.61; Nurses 3.90 ± 1.66; *p* = 0.18). (Media Use) Use of media formats (D 4.06 ± 0.40; N 4.23 ± 0.56; *p* = 0.27). (Enviroment) Learning environment (D 4.43 ± 0.50; N 4.20 ± 0.76; *p* = 0.32). (Independant Use) Independent use of learned content (D 5.18 ± 1.23; N 3.00 ± 1.41; *p* = 0.008). Six-point-scale 1 = strongly disagree, 6 = strongly agree; exception at media use with five-point-scale, 1 = strongly disagree, 5 = strongly agree.

Survey questions on specific topics were grouped for analysis. Participants were asked about subjective changes in sonographic competence, use of different media formats, learning environment, and independent use of learned content (see Figure 3). All participants reported subjective learning progress as a result of the training (Figure 3; Skill acquisition). The combined mean value is 4.15 with a standard deviation of 1.28. The questions of the validated GEKo assessment address issues of broadened subject knowledge and the formation of contexts (Table 1). The use of different media formats was also rated very positively by the participants, especially considering the five-point scale (mean = 4.05, SD = 0.53). There was widespread agreement that the media-supported preparation promoted individual learning processes and improved the assessment of personal learning progress. The practical relevance and the organization of the course content have also been improved (Figure 3; Media use). The third group of questionnaire items focused on the learning environment. Here, we recorded how the participants perceived the temporal and intellectual scope of the training and how they rated the personal supervision. Subjective under- or overload and compatibility with shift work were also addressed (mean = 4.35, SD = 0.59; see Figure 3; Environment). Finally, we asked about future independent use of the learned skills and abilities to independently assess sonographic findings of performed examinations (mean = 4.26, SD = 1.62; see Figure 3; Independent use). Participants were given the opportunity of additional comments to their responses in free text. Comments from participants who felt confident in their ability to independently apply what they had learned in the future included very positive feedback for the (personal) training and the clear examination procedure. The main reasons given for low confidence were a lack of time in the daily work routine and lack of individual supervision.

## 4. Discussion

### 4.1. General conclusions

In this study, we present a comprehensive approach to teaching new skills during ongoing ward routines. In doing so, we had to consider aggravating initial conditions such as shift work or high staff workload to introduce a new medical intervention into existing work routines. This led to a multimodal structure that addressed these circumstances through increased flexibility and asynchronous access to the educational content. In addition, we used modern media formats such as YouTube videos, quizzes, or pocket cards to increase acceptance. Through this study, we were able to fully train 32 members of our staff, who in turn applied the learned sonography scheme to 550 patients.

Despite repeated invitations, the response rate to the questionnaire remained low, which limits the significance of the results in terms of content. Nevertheless, the data presented confirms the learning success of the training. Participants felt more confident using and implementing the RADiUS protocol as a result of the interventions. Comparison of competency assessments showed significant gains in both general and thoracic sonography for both physicians and nurses. In addition, participants rated the use of multiple media formats as beneficial, both for personal learning and for compatibility with working conditions. Here, the survey results for media use are more clearly positive than for skill acquisition (see Figure 3), especially since the Likert scale for media use was only five instead of six levels. The complementary use of asynchronous teaching materials not only improved the learning process, but also provided a low-threshold and constant availability of relevant information. In addition to the high demand for the pocket cards, the digital content provided (videos, manual) was viewed almost 200 times. This indicates a repeated use and a possible expansion of the user group of the provided resources. In addition, the complexity of the content taught, and the training capacity provided were rated positively by most participants (see Figure 3; Environment). However, some individuals mentioned a lack of opportunities for individual training.

Heavy workloads and limitations due to shift work were among the most frequently cited reasons. This opinion was also reflected in the form of free text (“Too little practice, no time to do this regularly,” “I would like more time and specialized personnel to be able to apply my acquired knowledge more consistently in everyday life”), mostly expressed by nurses. These experiences during our study illustrate the considerable need for training of non-educated personnel in terms of sonography. In addition to the numerous educational references offered, live and individual interprofessional teaching remains the essential component for sufficient knowledge transfer. These results are underlined by the heterogeneous indications regarding the independent continuation of thoracic sonography (see Figure 3; Independent use). A large proportion of respondents indicated that they continued to apply what they had learned. However, some individuals disagreed. This is even more the case when asked about the interpretation of sonographic findings. While the differences between physicians and nurses were not significantly different for the other items, here the deviation within the small sample is significant. Overall, physicians were willing to apply the newly learned knowledge independently, whereas most nurses disagreed. This indicates that nurses require delegation and time to perform sonography.

Overall, however, we were able to show that a multimodal teaching process including digital media is successful in improving the skills of both nurses and physicians in thoracic ultrasound. An effective teaching program, with a short personal teaching effort of 30–120 min, can be established in emergency departments. We plan to continue this concept and extend it to other areas of emergency ultrasound diagnostics.

### 4.2. Study limitations

The statistical power of the survey is limited by the fact that it was conducted only after the intervention. An evaluation based on two questionnaires, one before and one after the training, would have been required to accurately determine the level of training of the medical personnel involved. Another limitation is the low response rate to the survey, since participation was voluntary. This limits the significance of the results, mandatory participation would have strengthened the statistical validity. In addition, a selection bias is likely. As the participation of the ward staff in the training was voluntary, it can be assumed that the motivation of the participants was higher and therefore interested participants were more likely to experience the intervention positively. The results may also be influenced by young participants with rather limited experience in emergency medicine.

### 4.3. Overall conclusion

Despite some limitations in our initial study on the development of a learning model for POCUS, we conclude that our established learning model is time- and resource-efficient for emergency departments. It can effectively enhance the implementation of Point-of-Care Sonography in these settings.

## Data availability statement

The original contributions presented in the study are included in the article/supplementary material, further inquiries can be directed to the corresponding author.

## Ethics statement

The studies involving human participants were reviewed and approved by the ethics committee of the University of Heidelberg. The patients/participants provided their written informed consent to participate in this study.

## Author contributions

TS, AK, MM, and MW contributed to the conception and design of the study. AK, MO, and SS assisted in the collection of data. MW and TS performed the statistical analysis. MW, TS, AK, and MO wrote the manuscript. All authors contributed to the article and approved the submitted version.

## Conflict of interest

The authors declare that the research was conducted in the absence of any commercial or financial relationships that could be construed as a potential conflict of interest.

## Publisher’s note

All claims expressed in this article are solely those of the authors and do not necessarily represent those of their affiliated organizations, or those of the publisher, the editors and the reviewers. Any product that may be evaluated in this article, or claim that may be made by its manufacturer, is not guaranteed or endorsed by the publisher.
